# MulNet: a scalable framework for reconstructing intra- and intercellular signaling networks from bulk and single-cell RNA-seq data

**DOI:** 10.1093/bib/bbaf081

**Published:** 2025-03-17

**Authors:** Mingfei Han, Xiaoqing Chen, Xiao Li, Jie Ma, Tao Chen, Chunyuan Yang, Juan Wang, Yingxing Li, Wenting Guo, Yunping Zhu

**Affiliations:** State Key Laboratory of Medical Proteomics, Beijing Proteome Research Center, National Center for Protein Sciences (Beijing), Beijing Institute of Lifeomics, No. 38, Life Science Park Road, Changping District, Beijing 102206, China; State Key Laboratory of Medical Proteomics, Beijing Proteome Research Center, National Center for Protein Sciences (Beijing), Beijing Institute of Lifeomics, No. 38, Life Science Park Road, Changping District, Beijing 102206, China; State Key Laboratory of Medical Proteomics, Beijing Proteome Research Center, National Center for Protein Sciences (Beijing), Beijing Institute of Lifeomics, No. 38, Life Science Park Road, Changping District, Beijing 102206, China; State Key Laboratory of Medical Proteomics, Beijing Proteome Research Center, National Center for Protein Sciences (Beijing), Beijing Institute of Lifeomics, No. 38, Life Science Park Road, Changping District, Beijing 102206, China; State Key Laboratory of Medical Proteomics, Beijing Proteome Research Center, National Center for Protein Sciences (Beijing), Beijing Institute of Lifeomics, No. 38, Life Science Park Road, Changping District, Beijing 102206, China; State Key Laboratory of Medical Proteomics, Beijing Proteome Research Center, National Center for Protein Sciences (Beijing), Beijing Institute of Lifeomics, No. 38, Life Science Park Road, Changping District, Beijing 102206, China; State Key Laboratory of Medical Proteomics, Beijing Proteome Research Center, National Center for Protein Sciences (Beijing), Beijing Institute of Lifeomics, No. 38, Life Science Park Road, Changping District, Beijing 102206, China; Central Research Laboratory, Peking Union Medical College Hospital, Chinese Academy of Medical Sciences and Peking Union Medical College, No. 1 Shuaifuyuan Wangfujing Dongcheng District, Beijing 100730, China; Central Research Laboratory, Peking Union Medical College Hospital, Chinese Academy of Medical Sciences and Peking Union Medical College, No. 1 Shuaifuyuan Wangfujing Dongcheng District, Beijing 100730, China; State Key Laboratory of Medical Proteomics, Beijing Proteome Research Center, National Center for Protein Sciences (Beijing), Beijing Institute of Lifeomics, No. 38, Life Science Park Road, Changping District, Beijing 102206, China

**Keywords:** multilayer network, reinforcement learning, gene regulation, cancer regulators

## Abstract

Gene expression involves complex interactions between DNA, RNA, proteins, and small molecules. However, most existing molecular networks are built on limited interaction types, resulting in a fragmented understanding of gene regulation. Here, we present MulNet, a framework that organizes diverse molecular interactions underlying gene expression data into a scalable multilayer network. Additionally, MulNet can accurately identify gene modules and key regulators within this network. When applied across diverse cancer datasets, MulNet outperformed state-of-the-art methods in identifying biologically relevant modules. MulNet analysis of RNA-seq data from colon cancer revealed numerous well-established cancer regulators and a promising new therapeutic target, miR-8485, along with several downstream pathways it governs to inhibit tumor growth. MulNet analysis of single-cell RNA-seq data from head and neck cancer revealed intricate communication networks between fibroblasts and malignant cells mediated by transcription factors and cytokines. Overall, MulNet enables high-resolution reconstruction of intra- and intercellular communication from both bulk and single-cell data. The MulNet code and application are available at https://github.com/free1234hm/MulNet.

## Introduction

A crucial aspect of analyzing genome-wide gene expression data is the inference of gene regulatory networks (GRNs) [1, 2]. Pioneering methods, for example, can reconstruct tissue-specific GRNs using bulk RNA-seq data [3–5]. With the advancements in single-cell RNA sequencing (scRNA-seq) technology, new computational approaches have emerged to infer cell type-specific GRNs. These methods can be broadly categorized into probabilistic model–based approaches, deep learning methods, and reinforcement learning (RL) techniques. Probabilistic model–based methods include approaches that utilize models such as random forests [6], Bayesian networks [7], graphical models [8], and probabilistic matrix factorization [9]. Deep learning–based methods leverage architectures like convolutional neural networks [10–12] and graph neural networks [13, 14]. Meanwhile, RL methods frame GRN inference as a Markov decision process (MDP), where an agent interacts with the environment in discrete time steps by observing states, taking actions, and receiving rewards, aiming to identify an optimal policy that maximizes cumulative reward [15].

While GRNs are valuable for identifying key transcription factors (TFs) within specific tissues or cells, they overlook other important intra- and intercellular interactions, such as ligand–receptor interactions, protein–protein interactions (PPIs), and microRNA (miRNA)–messenger RNA (mRNA) interactions. Consequently, GRNs are limited in their ability to capture the full complexity of biological systems. Understanding how various interactions synergistically regulate gene expression remains a significant challenge.

**Figure 1:**
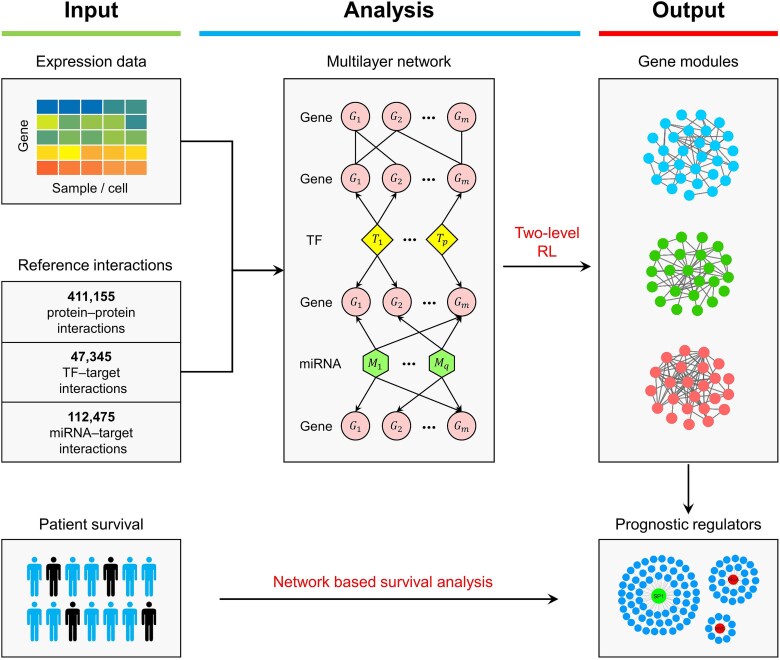
Overview of the MulNet workflow. The input to MulNet includes a tissue- or cell-type-specific gene expression matrix along with reference interactions. MulNet first constructs a multilayer network by integrating the gene expression data with reference interactions and then identifies gene modules within this network using hierarchical reinforcement learning. By incorporating patient survival outcomes, MulNet can further identify prognostic regulators and their downstream targets.

Here, we present MulNet (Multilayer Network), a framework for reconstructing multi-interaction networks from gene expression data. MulNet consists of three main steps (Fig. 1). First, it constructs a multilayer network (MLN) by integrating gene expression data with reference interactions. Users can upload their own interaction data or use our curated collection of human reference interactions from 12 databases. Second, MulNet identifies gene modules within the multilayer network using a two-level RL framework. Third, by incorporating patient survival outcomes, MulNet can predict prognostic regulators and their downstream targets through network-based survival analysis.

Applying MulNet to bulk and single-cell RNA-seq data across diverse cancer types, we demonstrated that its two-level RL framework outperforms state-of-the-art methods in identifying biologically relevant modules. Furthermore, the network-based survival analysis revealed numerous cancer-related TFs and miRNAs that evaded detection by conventional survival analysis. These findings highlight the two key innovations of MulNet—the two-level RL framework and network-based survival analysis—which significantly improve gene module detection and the identification of prognostic regulators.

Two case studies highlight MulNet’s potential in cancer research. First, MulNet analysis of RNA-seq data from colon adenocarcinoma identified numerous well-established cancer regulators and a promising therapeutic target, miR-8485, along with several downstream pathways it modulates to inhibit tumor growth. Second, MulNet analysis of scRNA-seq data from head and neck cancer revealed a complex signaling network between fibroblasts and malignant cells. This network demonstrates how TFs within fibroblasts regulate the expression of ligand molecules that, once secreted, bind to receptors on tumor cells, triggering downstream transcriptional responses.

## Materials and methods

### Construction of multilayer networks

MulNet requires two types of input data: tissue- or cell-type-specific expression data $M$ and $k$ reference interaction datasets $\left\{{N}_1,\dots{N}_k\right\}$. Users can upload their own interaction data or use a curated library of human reference interactions. This library includes 411 155 PPIs from STRING, BIOGRID, DIP, HuRI, and MINT; 47 345 TF–target interactions from hTFtarget, RegNetwork, TFLink, and TRRUST; and 112 475 miRNA–target interactions from miRDB, miRTarBase, and RegNetwork. To ensure reliability, we retained only interactions supported by at least two independent databases.

**Figure 2:**
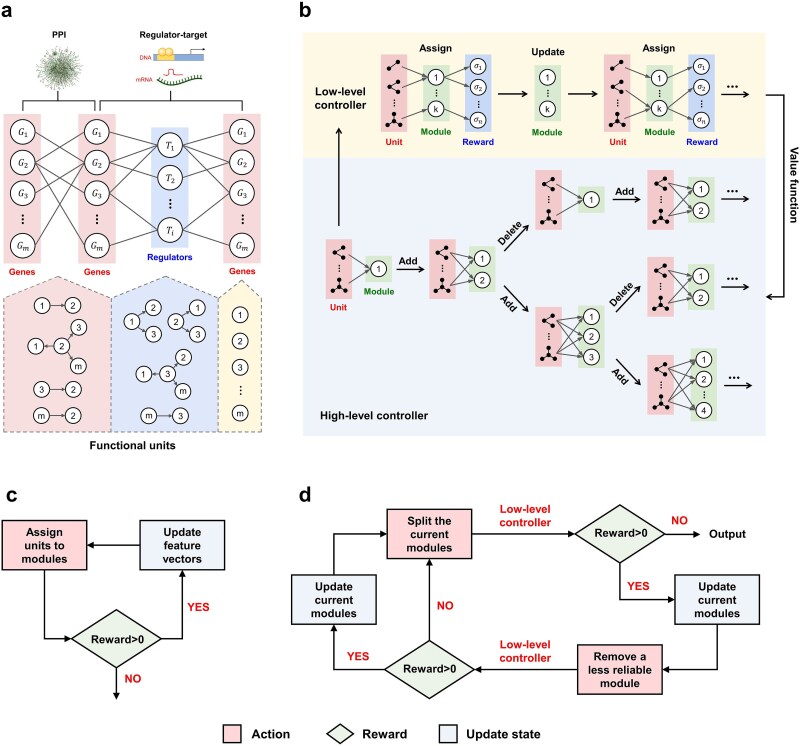
Multilayer network construction and gene module detection. (a) Architecture of the multilayer network model. The model supports scalable numbers of gene and functional layers. The analysis involves partitioning gene and functional layers into functional units, which are then organized into signaling modules. (b) Schematic of the two-level RL framework. The algorithm consists of a high-level controller that identifies new modules and a low-level controller that assigns functional units to existing modules. (c) Flowchart of the low-level controller. (d) Flowchart of the high-level controller.

By integrating these data, MulNet constructs a ($k+1$)-layer network. Each layer includes all genes present in $M$. Nodes in adjacent layers are linked by specific interactions within ${N}_1$ to ${N}_k$. However, the relatively low expression levels of some gene expression regulators result in many meaningful interactions being excluded from the network. To address this, we added functional layers (TFs and miRNAs in Fig. 2) to encompass important functional molecules that may not be present in $M$ but whose effects on downstream genes are included in the reference interaction data.

### Prepossessing of multilayer networks

MulNet preprocesses multilayer networks in two steps. The first step is to remove all functional layers and establish direct connections between the gene layers on both sides of the removed functional layers, thereby rewiring regulator–target interactions into coregulatory relationships between target genes. Subsequently, MulNet weights the network connections using both prior confidence scores (optional) and the probabilities of expression correlations between interacting genes (required), thus reducing the impact of interactions with low confidence or inconsistencies with the expression data. Given a gene ${G}_0$ and its neighbors in the right gene layer (${G}_1,\dots, {G}_k$), the weights of the connections between ${G}_0$ and ${G}_i$ ($i\in \left[1,k\right]$) are calculated using the softmax function:


$$ {W}_{0,i}=\frac{e^{S_{0,i}\times{P}_{0,i}}}{\sum_{j=1}^k{e}^{S_{0,i}\times{P}_{0,j}}} $$

where ${S}_{0,i}$ represents the prior confidence score between ${G}_0$ and ${G}_i$ in user-uploaded interaction data (normalized to $\left[0,1\right]$), which is either obtained from public databases or defined by the user. This score is set to 1 if no confidence scores are provided. ${P}_{0,i}$ represents the probability of correlation between ${G}_0$ and ${G}_i$ (details in Supplementary Method 1 and Supplementary Fig. S1).

### Identification of functional units

In the rewired and weighted MLN, a single gene can act as multiple functional units, either independently or collaborating with partner molecules. For example, ${G}_1$ in Fig. 2a forms three functional units: itself, together with interacting partner ${G}_2$, and together with coregulatory partners ${G}_2$ and ${G}_3$. This is consistent with the combinatorial nature of gene expression regulation. A functional unit consisting of a single gene is referred to as a single-gene functional unit, while a functional unit comprising a gene and its interacting partners is termed a combined functional unit.

From an expression matrix containing $m$ genes and $n$ samples, $m$ single-gene functional units and an unknown number of combined functional units can be derived. Each functional unit is characterized by an $n$-dimensional expression pattern. The expression pattern of a single-gene functional unit can be directly extracted from the expression matrix. The expression pattern of a combined functional unit, in which ${G}_0$ interacts with (${G}_1,\dots, {G}_k$), is calculated as ${\sum}_{i=1}^kW\left({G}_0,{G}_i\right)\times{V}_i$, where $W\left({G}_0,{G}_i\right)$ represents the connection weight between ${G}_0$ and ${G}_i$ (detailed in Prepossessing of Multilayer Networks) and ${V}_i=\left({v}_{i,1},\dots, {v}_{i,n}\right)$ represents the *z*-score normalized expression pattern of ${G}_i$ ($i\in \left[1,k\right]$). The core idea of a combined functional unit is to represent a gene’s expression pattern as a weighted average of the expression patterns of its interacting partners.

### Identification of gene modules

Reference interactions are often not specific to the gene expression data being analyzed, which can result in false-positive functional units. To mitigate this, we utilized a two-level RL algorithm to group functional units into data-driven functional modules. This algorithm employs two controllers: a high-level controller that identifies new modules and a low-level controller that assigns functional units to existing modules (Fig. 2b). Each controller operates within its own MDP, where an agent interacts with the environment in discrete time steps by observing states, taking actions, and receiving rewards.

#### The low-level controller

At each time step, the low-level controller assigns each functional unit to the module with the highest probability of correlation (Fig. 2c). For a single-gene functional unit, the probability of correlation with $M$ is determined as described in Supplementary Method 1. For a combined functional unit $u$ consisting of three genes $\left\{{g}_1,{g}_2,{g}_3\right\}$, where ${g}_1$ is connected to ${g}_2$ and ${g}_3$ with weights ${w}_{1,2}$ and ${w}_{1,3}$, respectively, the probability of correlation between $u$ and $M$ is calculated as ${w}_{1,2}\times P\left({g}_2,M\right)+{w}_{1,3}\times P\left({g}_3,M\right)$, where $P\left(g,M\right)$ represents the probability of correlation between gene $g$ and module $M$. The cumulative probability of assigning all units to the most relevant module is calculated as follows:


$$ CP={\sum}_{i=1}^N\underset{j\in \left[1,k\right]}{\max }P\left({u}_i,{M}_j\right) $$

After assigning all functional units to their most relevant modules, the agent updates the feature vector of each module and proceeds to the next assignment round. A module’s feature vector is calculated as the average expression pattern of the functional units assigned to it. The reward for the $t$-th assignment is calculated as $R\left({s}_t|{s}_{t-1}\right)={CP}_t-{CP}_{t-1}$. When $R\le 0$, the agent reaches a terminal state and returns the optimal $CP$ to the high-level controller, serving as the value function for the high-level controller’s previous action.

#### The high-level controller

Starting with an initial module containing all functional units, the high-level controller alternates between two actions: split the current modules and removing a less reliable module (Fig. 2d).

Given a state ${s}_n$ with $n$ modules, the goal of splitting modules is to determine the optimal way to add a new module to ${s}_n$. Consider a module $M$ as an example. First, the correlation scores between $M$ and its functional units are calculated. For a single-gene functional unit $g$, the correlation score with $M$ is defined as the absolute Pearson correlation coefficient between the expression pattern of $g$ and the feature vector of $M$. For a combined functional unit $u$ consisting of three genes $\left\{{g}_1,{g}_2,{g}_3\right\}$, where ${g}_1$ is connected to ${g}_2$ and ${g}_3$ with weights ${w}_{1,2}$ and ${w}_{1,3}$, respectively, the correlation score between $u$ and $M$ is calculated as ${w}_{1,2}\times \left|r\left({g}_2,M\right)\right|+{w}_{1,3}\times r\left({g}_3,M\right)$, where $r\left(g,M\right)$ represents the Pearson correlation coefficient between the expression pattern of $g$ and the feature vector of $M$. Finally, $M$ is split into two submodules: one containing functional units with correlation scores >0.4 and the other containing functional units with correlation scores <0.4.

Splitting each module of ${s}_n$ creates $n$ temporary states $\left\{{s^1}_{n+1},\dots, {s^n}_{n+1}\right\}$. The reward of each temporary state is calculated by the low-level controller (details in The Low-level Controller section). The best reward is calculated as $R\left({s}_{n+1}|{s}_n\right)=\gamma \times \mathit{\max}\left\{V\left({s}_{n+1}\right)\right\}-V\left({s}_n\right)$, where $V$ represents the value function returned by the low-level controller, $\mathit{\max}\left\{V\left({s}_{n+1}\right)\right\}$ represents the optimal value function for different splits, and $\gamma$ is a discount factor (0.995 by default) to limit overfitting. If $R\left({s}_{n+1}|{s}_n\right)>0$, the agent will update ${s}_n$ to ${s}_{n+1}$ based on the action with the best reward. Otherwise, the agent stops the training process and outputs the current modules.

To remove a module from state ${s}_n$ ($n>1$), the agent removes each module of ${s}_n$ to create $n$ temporary states $\left\{{s^1}_{n-1},\dots, {s^n}_{n-1}\right\}$. The reward of this action is $R\left({s}_{n-1}^{\prime }|{s}_{n-1}\right)=\mathit{\max}\left\{V\left({s}_{n-1}^{\prime}\right)\right\}-V\left({s}_{n-1}\right)$, where $\mathit{\max}\left\{V\left({s}_{n-1}^{\prime}\right)\right\}$ represents the optimal value function for removing different modules, and $V\left({s}_{n-1}\right)$ represents the previous value function of state ${s}_{n-1}$ stored in the system. If the reward is greater than 0, the agent will update ${s}_n$ to the optimal ${s}_{n-1}^{\prime }$ and store the value function of ${s}_{n-1}^{\prime }$ in the system. The removal action continuously optimizes the previous states by backtracking, thereby avoiding local optima.

#### Network construction

After both controllers terminate, each functional unit will be assigned to the module exhibiting the highest correlation probability. This assignment allows for module overlap, as genes can function as distinct functional units, operating autonomously or in conjunction with other genes, and therefore can be assigned to multiple modules. Finally, genes belonging to the same module are interconnected via various interactions extracted from the MLN, forming a biologically relevant multi-interaction network. For genes lacking known interactions within the MLN, coexpression relationships are utilized to establish connections. All gene modules can be assembled into a genome-wide multi-interaction network.

### Network-based survival analysis

**Figure 3:**
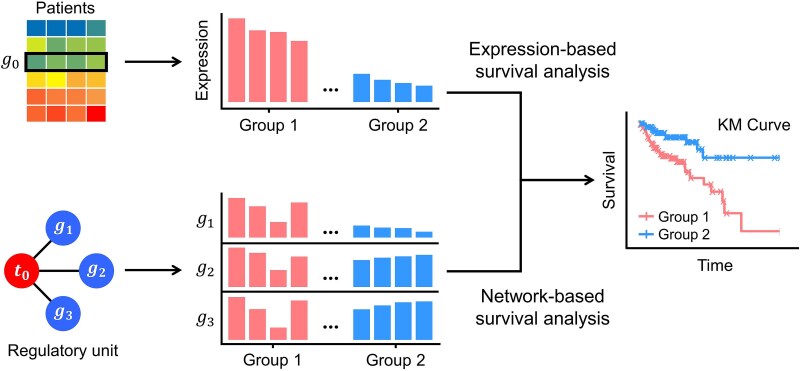
Schematic diagram of two survival analysis methods. Conventional expression-based survival analysis (the upper path) ranks patients based on the expression of _0 and estimates the survival difference between the top- and bottom-ranked patients. Network-based survival analysis (the lower path) ranks patients based on the expression similarity between _1 and its coregulatory partners.

By integrating clinical outcomes such as overall survival, MulNet can further identify prognostic regulators and their downstream targets. Due to the generally low expression of regulators, especially miRNAs, most existing tools [16–18] that investigate the correlation between individual gene expression and patient survival (the upper path in Fig. 3) are limited in identifying prognostic regulators.

In this study, we addressed this limitation by leveraging the coexpression properties of coregulated genes. Taking the lower path in Fig. 3 as an example, three coregulated genes (${g}_1$, ${g}_2$ and ${g}_3$) exhibited similar expression patterns in patient group 1 when their coregulator ${t}_0$ was functioning well (red bars). When ${t}_0$ lost control of ${g}_1$ in patient group 2, ${g}_1$ lost similarity with the other two genes (blue bars). A significant survival difference between patient groups 1 and 2 indicated that the regulatory relationship between ${t}_0$ and ${g}_1$ is associated with patient survival.

To implement the aforementioned process, we first applied z-score normalization to ensure the same weight of each gene. The normalized expression patterns of gene ${g}_i$ ($i\in \left[1,3\right]$) are denoted as ${V}_i=\left\{{v}_{i,1},\dots, {v}_{i,n}\right\}$. Subsequently, we calculated the expression similarity between ${g}_1$ and its coregulatory partners in each patient as:


$$ {S}_j={\left({v}_{1,j}-{v}_{2,j}\right)}^2+{\left({v}_{1,j}-{v}_{3,j}\right)}^2 $$

Patients were then ranked based on the expression similarity scores ${S}_j$ ($j\in \left[1,n\right]$). The log-rank test was employed to compare survival curves between the top- and bottom-ranked patients.

### User-friendly tool

We developed MulNet, a gene expression analysis tool that integrates multilayer network construction, gene module detection, and prognostic regulator identification. MulNet provides an intuitive user experience with interactive tables, heatmaps, and network diagrams, enabling efficient exploration of gene modules and prognostic networks (Supplementary Fig. S2). MulNet is a platform-independent application implemented in Java. It requires Java 1.5 or later to run and has been tested for compatibility on both Windows and Linux operating systems (see MulNet User Manual for details).

### Limitation of MulNet

MulNet provides an effective multilayer network model for integrating gene expression data with various molecular interactions. However, a notable limitation of this approach is its applicability predominantly to species such as humans and mice, which benefit from extensive reference interaction data. Conversely, MulNet is less effective for analyzing gene expression data from species such as *Escherichia coli* and yeast, where reference interactions are insufficient. We anticipate that this limitation will be mitigated in the future as more molecular interactions are discovered and experimentally validated.

## Results

### Module detection benchmark

To evaluate the effectiveness of MulNet, we benchmarked it against top-performing methods identified in two previous studies [19, 20]. These included eight methods designed for bulk RNA-seq analysis (ICA [21], WGCNA [22], FLAME [23], spectral clustering [24], Dclust [25], spectral biclustering [26], MERLIN [27], and GENIE3 [3]) and six methods tailored for scRNA-seq analysis (SCENIC [6], SCIMITAR [28], NLNET [29], SCODE [30], PIDC [31], and LEAP [32]).

**Figure 4:**
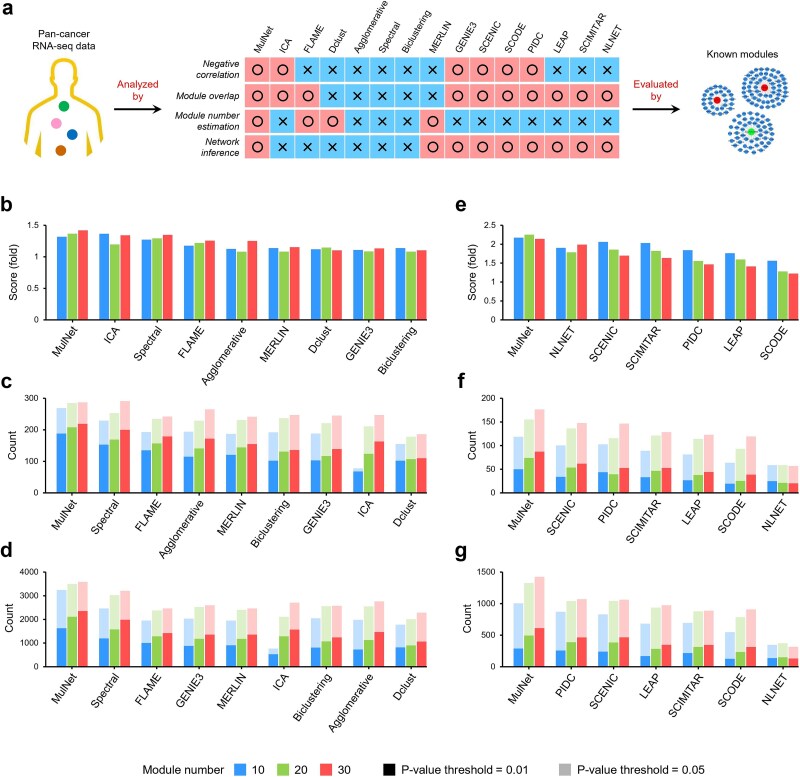
Method evaluation. (a) Overview of the evaluation pipeline. (b–d) Performance metrics comparing MulNet with eight bulk RNA-seq analysis methods using TCGA pan-cancer data. (b) Scores reflecting the agreement between predicted modules and known modules. (c) Number of significantly enriched KEGG pathways at *P*-value thresholds of .01 (dark bars) and .05 (light bars). (d) Number of significantly enriched GO terms. (e–g) Performance metrics comparing MulNet with six scRNA-seq analysis methods on single-cell RNA-seq datasets spanning 10 cancer types. Metrics were averaged across the 10 datasets for a comprehensive assessment.

MulNet stands out among these methods by addressing several challenges (Fig. 4a). First, it identifies both positively and negatively correlated genes within the same module, reflecting the inherent duality of positive and negative regulatory relationships within biological systems [33]. Second, it allows for module overlap, consistent with the highly combinatorial nature of gene regulation [34]. Third, it can automatically estimate the optimal number of modules. Finally, it models the regulatory and functional relationships among molecules through multi-interaction networks.

We applied MulNet and the eight bulk RNA-seq analysis methods to The Cancer Genome Atlas (TCGA) pan-cancer data to identify gene modules (details in Supplementary Methods 2.2). Module quality was assessed by comparing the identified modules against a set of known modules (see Supplementary Methods 2.1). The processed TCGA data and the known modules were created by Saelens *et al*. [19] and are publicly available on Zenodo (https://zenodo.org/records/1157938). The results demonstrated that MulNet outperformed all tested methods in retrieving biologically relevant modules (Fig. 4b). Furthermore, MulNet identified the highest number of enriched KEGG pathways and Gene Ontology (GO) terms, as shown in Fig. 4c and d.

The second benchmark study utilized human scRNA-seq datasets spanning 10 cancer types: breast, head and neck, prostate, colorectal, liver, pancreatic, lung, hematologic, kidney, and ovarian cancers. The preprocessed datasets were sourced from the Curated Cancer Cell Atlas (www.weizmann.ac.il/sites/3CA/) [35]. For each dataset, we constructed a malignant cell-specific expression matrix and applied MulNet alongside six other scRNA-seq analysis methods to identify gene modules. Detailed descriptions of the data processing and module detection steps are provided in Supplementary Methods 2.3.

The resulting gene modules were evaluated against known cancer modules identified by Gavish *et al*. [35], comprising 148 gene sets that are coordinately upregulated in cell subpopulations across 24 tumor types (available in MSigDB). Metrics were averaged across the 10 datasets for a comprehensive assessment. The results demonstrated that MulNet consistently outperformed other methods in identifying known modules and in KEGG and GO enrichment analyses (Fig. 4e–g). Overall, MulNet demonstrates superior performance in uncovering coregulated and functionally related genes from both bulk and single-cell RNA-seq data.

### Reconstructing intracellular signaling networks

**Figure 5:**
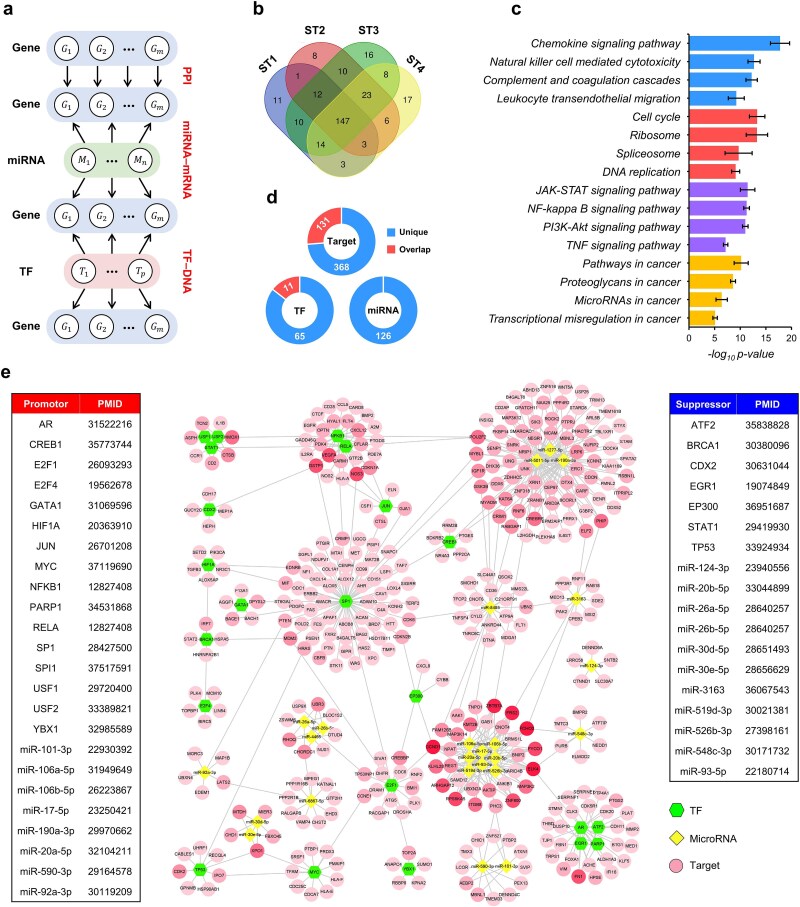
Case study of COAD. (a) Schematic of the COAD multilayer network, incorporating four gene layers and two functional layers. (b) Venn diagram of KEGG pathways identified in stage I–IV COAD. (c) Representative pathways shared across the four COAD stages. The bar graph shows the mean ± SD of the negative log10 of hypergeometric test *P*-values for different COAD stages. (d) The number of TFs, miRNAs, and target genes identified by network-based survival analysis. The “overlap” part represents elements whose individual expression levels are significantly correlated with overall survival. (e) COAD survival network in which each TF and miRNA regulates at least five survival genes. Boxes highlight regulators reported to promote or inhibit COAD, together with their PMIDs in PubMed.

To assess MulNet’s ability to extract biological insights from bulk gene expression data, we applied it to TCGA RNA-seq data from stage I to IV colon adenocarcinoma (COAD) patients (details in Supplementary Methods 3). This analysis yielded four stage-specific MLNs, each incorporating three types of intracellular interactions: PPIs, TF–DNA interactions, and miRNA–mRNA interactions (Fig. 5a). Two-level RL performed on these MLNs identified 27, 21, 25, and 32 gene modules for stage I–IV COAD, respectively (Supplementary Table S1). KEGG enrichment analysis revealed 201, 210, 240, and 221 significantly enriched pathways in these modules (Supplementary Table S2), with 147 pathways shared across all four COAD stages (Fig. 5b). These pathways encompass four well-established functional groups in cancer (Fig. 5c): cell proliferation, immune system, signal transduction, and cancer pathways.

We constructed a “consensus network” by integrating the gene modules identified across stage I–IV COAD (Supplementary Table S3). In this network, nodes are connected only if they consistently participate in the same module across all four stages. Network-based survival analysis of the consensus network revealed 76 TFs, 126 miRNAs, and 499 target genes associated with overall survival in COAD patients (Supplementary Table S4). Remarkably, 74% of these target genes, 85% of the TFs, and all the miRNAs evaded detection by conventional expression-based survival analysis (Fig. 5d), highlighting the limitations of solely depending on expression levels to uncover prognostic biomarkers. This is likely due to the generally low expression of regulators, especially miRNAs, and the potential for post-translational modifications and protein structure to influence binding affinity independently of expression levels.

Figure 5e depicts 23 TFs and 24 miRNAs that regulate at least five survival genes. Evidence from existing studies suggests that all these TFs and 19 miRNAs have well-established roles in colorectal cancer, including 24 markers of poor prognosis and 18 tumor suppressors (boxes in Fig. 5e). These results highlight the promising potential of network-based survival analysis for advancing cancer research. Among the five unreported miRNAs, we selected miR-8485, which independently regulates the largest number of survival genes, for further investigation.

### The role of miR-8485 in colon adenocarcinoma

**Figure 6:**
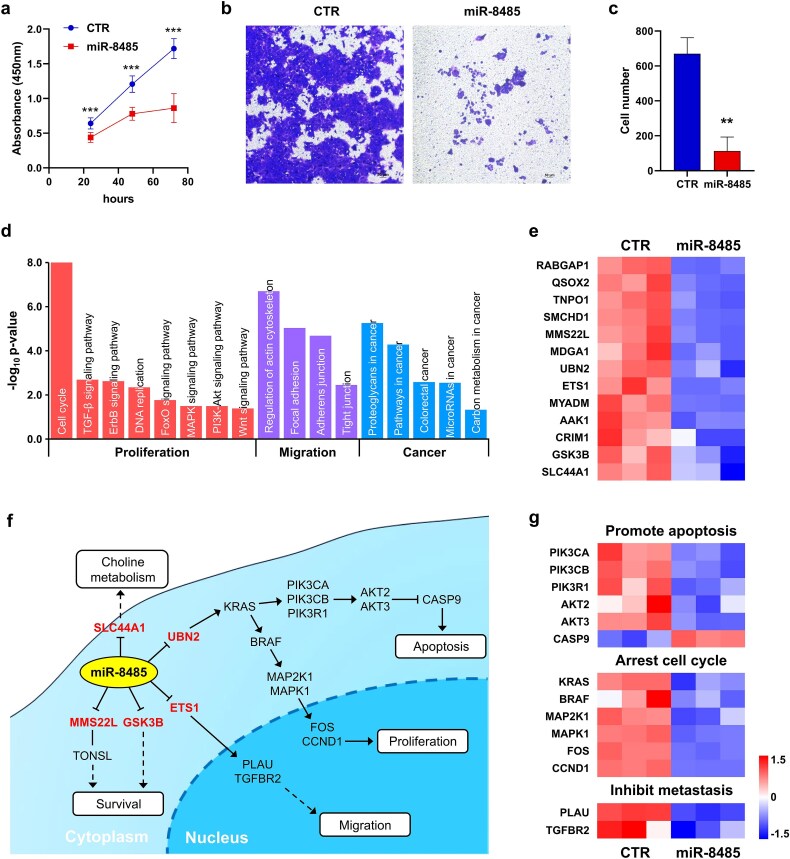
Role of miR-8485 in the HCT116 cell line. (a) Proliferation of HCT116 cells transfected with control (CTR) or miR-8485 mimics, as determined by CCK-8 assay. (b) Cell migration ability of the miR-8485 and CTR groups determined by transwell assays. (c) Statistical analysis of the number of migrated cells. (d) Representative pathways enriched in the genes downregulated in miR-8485-transfected HCT116 cells compared to CTR cells. (e) Heatmap of seven target genes of miR-8485 that were significantly downregulated upon miR-8485 overexpression. (f) Three pathways associated with the inhibitory effects of miR-8485 on COAD cells. (g) Heatmaps of some key genes within the three pathways.

We overexpressed miR-8485 in the HCT116 COAD cell line by transfecting miR-8485 mimics (details are provided in Supplementary Methods 5). CCK-8 assays (Fig. 6a) revealed that miR-8485 significantly inhibited the proliferation of HCT116 cells at 24, 48, and 72 h after transfection. Transwell migration assays (Fig. 6b and c) revealed that compared with control transfection, miR-8485 transfection significantly inhibited the migration ability of HCT116 cells. Additionally, we injected HCT116 COAD cells transfected with control or miR-8485 into nude mice (see Supplementary Methods 6 for details). The miR-8485-transfected group exhibited a dramatic decrease in tumor volume (Supplementary Fig. S3), indicating that miR-8485 significantly decreased the subcutaneous tumor formation rate (66% versus 100%) and tumor growth rate.

We further analyzed the whole transcriptome of miR-8485-transfected COAD cells to investigate the inhibitory mechanisms (details in Supplementary Methods 5.5). Differential expression analysis revealed 3810 upregulated and 4125 downregulated genes in miR-8485-transfected HCT116 COAD cells compared to the control group (Supplementary Fig. S4 and Supplementary Table S5). Enrichment analysis revealed 12 KEGG pathways significantly enriched in the upregulated genes and 62 significantly enriched in the downregulated genes (Supplementary Fig. S5 and Supplementary Table S6). We found that the downregulated pathways were closely associated with cell proliferation, cell migration, and cancer progression (Fig. 6d), demonstrating that miR-8485 plays a tumor suppressor role in COAD.

To elucidate the pathways directly influenced by miR-8485 transfection, we analyzed the expression of 26 miR-8485 target genes in the COAD survival network. RNA-seq analysis revealed that miR-8485 transfection resulted in significant downregulation of 13 target genes (Fig. 6e and Supplementary Table S7). Five of these genes have well-established roles in COAD development and progression (Fig. 6f). First, miR-8485-mediated downregulation of UBN2 inhibits cell proliferation via the RAS/MAPK pathway [36] and induces apoptosis through the RAS/PI3K/AKT pathway [37]. Second, miR-8485 suppresses COAD metastasis by targeting ETS1 [38], a TF that activates the expression of several extracellular matrix remodeling genes, such as TGFBR2 [39] and PLAU [40]. Third, the MMS22L-TONSL complex and GSK3B, which are downregulated by miR-8485, play crucial roles in maintaining COAD cell survival [41]. Finally, miR-8485 downregulates SLC44A1, a key choline transporter, inhibiting tumor progression by reducing choline uptake [42]. RNA-seq analysis revealed that the expression of key genes involved in the aforementioned pathways was significantly altered in the predicted directions following miR-8485 overexpression (Fig. 6g and Supplementary Table S8). These findings confirm the functional presence of these pathways in miR-8485-mediated COAD suppression.

In conclusion, miR-8485-mediated downregulation of UBN2, ETS1, MMS22L, GSK3B, and SLC44A1 inhibits tumor progression through mechanisms involving cell cycle arrest, metastasis inhibition, apoptosis induction, and choline metabolism. Further preclinical and clinical studies are warranted to substantiate these findings and pave the way for the development of miR-8485-based therapeutic strategies for COAD.

### Reconstructing intercellular communication based on single-cell data

**Figure 7:**
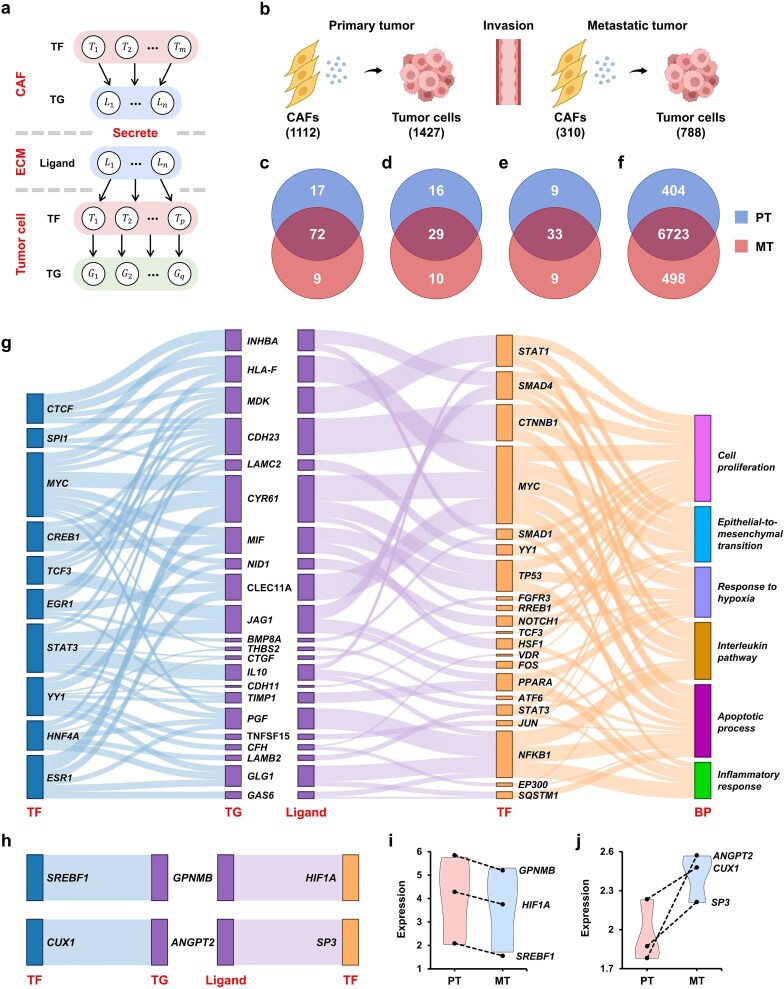
Case study of HNSCC. (a) Schematic of the fibroblast-tumor cell crosstalk network. (b) The numbers of CAFs and tumor cells in primary and metastatic tumors, respectively. (c–f) Venn diagrams of CAF TFs, secreted ligands, tumor cell TFs, and tumor cell target genes identified in primary and metastatic tumors. (g) Sankey diagram of CAF TFs, secreted ligands, and tumor cell TFs shared by primary and metastatic tumors. The rightmost layer displays biological processes enriched in the tumor cell TFs. Representative biological processes enriched in tumor cell TFs are shown on the right. (h) Sankey diagram of CAF TFs, secreted ligands, and tumor cell TFs unique to primary (upper path) and metastatic (lower path) tumors. (i) SREBF1 and GPNMB expression in primary and metastatic tumor fibroblasts and HIF1A expression in primary and metastatic tumor cells. (j) CUX1 and ANGPT2 expression in primary and metastatic tumor fibroblasts and SP3 expression in primary and metastatic tumor cells.

In the second case study, we applied MulNet to single-cell RNA-seq data from head and neck squamous cell carcinoma (HNSCC) tumors [43] to reconstruct fibroblast–malignant cell crosstalk. First, leveraging cell-specific gene expression data, we constructed two distinct molecular networks, a TF–target network for cancer-associated fibroblasts (CAFs) and a multi-interaction network for tumor cells, incorporating both ligand–TF interactions and TF–target interactions (details in Supplementary Methods 4). Subsequently, we assembled these two networks into a comprehensive CAF–tumor cell crosstalk network (Fig. 7a). This network reveals how TFs within CAFs regulate the expression of ligand molecules that, upon secretion, bind to receptors on tumor cells and trigger downstream transcriptional responses.

We separately analyzed primary and metastatic HNSCC tumors (Fig. 7b), reconstructing the CAF-tumor cell network for each (Supplementary Tables S9 and S10). Comparison of the primary and metastatic networks revealed shared TFs and ligand molecules between primary and metastatic tumors, including 72 CAF TFs (Fig. 7c), 29 secreted ligands (Fig. 7d), 33 tumor cell TFs (Fig. 6e), and 6723 tumor cell target genes (Fig. 7f). Fig. 7g depicts a subnetwork of these shared elements, where 10 CAF TFs (blue bars, each regulating at least five shared ligands) regulate the expression of 22 ligands (purple bars) and further affect 31 TFs in tumor cells (orange bars). Evidence from existing studies suggests that all these TFs and ligands have well-established roles in HNSCC or other cancers (details in Supplementary Table S11). Additionally, GO enrichment analysis of tumor cell TFs revealed their involvement in several cancer-related biological processes, including cell proliferation, epithelial-to-mesenchymal transition, response to hypoxia, interleukin pathway, apoptotic process, and inflammatory response (Fig. 7g and Supplementary Table S12). In summary, the signaling network shared by primary and metastatic tumors reveals several critical functions of fibroblasts in shaping tumor cell behavior [44].

Analysis of TFs and ligands unique to primary and metastatic tumors (Fig. 7c–e) revealed distinct signaling pathways active in each tumor type. The primary pathway comprises the CAF TF SREBF1, transmembrane glycoprotein NMB (GPNMB), and the tumor cell TF HIF1A (the upper path in Fig. 7h). Previous studies have proven that SREBF1, GPNMB, and HIF1A can promote primary tumor growth in a variety of cancers [45–47]. The metastatic pathway comprises the CAF TF CUX1, angiopoietin 2 (ANGPT2), and the tumor cell TF SP3 (the lower path in Fig. 7h). Both CUX1 and SP3 are key regulators of tumor aggressiveness [48, 49]. Additionally, ANGPT2 can regulate lymphatic vessel development [50], suggesting its crucial role in lymph node metastatic tumors.

Subsequently, we performed differential expression analysis on the aforementioned genes using single-cell RNA-seq data from Puram *et al*. This analysis revealed that SREBF1 and GPNMB were downregulated in metastatic tumor fibroblasts compared to their primary tumor counterparts, and HIF1A was downregulated in metastatic tumor cells compared to primary tumor cells (Fig. 7i). In contrast, CUX1 and ANGPT2 were upregulated in metastatic fibroblasts, and SP3 was upregulated in metastatic tumor cells relative to their expression in the primary tumor (Fig. 7j). These results suggest strong agreement between the observed gene expression changes and the predictions generated by MulNet. Additionally, MulNet can reveal potential regulators whose differential expression may not have reached statistical significance (e.g. SREBF1 and CUX1).

## Discussion

Despite advances in experimental and computational techniques, which enable increasingly accurate predictions of molecular interactions, the numerous and varied interactions in public databases remain largely untapped. In this study, MulNet offers a promising solution by integrating gene expression data with various molecular interaction databases, aiming to reveal how known or predicted interactions synergistically regulate gene expression. The flexibility of MulNet in integrating user-specified interactions could support its broad application in both bulk and single-cell omics studies.

**Key Points**

- MulNet provides a systematic gene expression analysis framework that involves multilayer network construction, gene module detection, and prognostic biomarker discovery.
- MulNet outperforms state-of-the-art methods in identifying biologically relevant modules and novel cancer regulators.
- MulNet enables high-resolution reconstruction of intra- and intercellular communication from both bulk and single-cell data.

## Supplementary Material

Supplementary_Materials_bbaf081

Supplementary_Tables_bbaf081

## Data Availability

For method evaluation, the TCGA pan-cancer datasets were obtained from Zenodo (https://zenodo.org/records/1157938). The scRNA-seq data across 10 cancer types were sourced from the Curated Cancer Cell Atlas (www.weizmann.ac.il/sites/3CA/). For the case studies, the COAD RNA-seq data were downloaded from the UCSC Xena browser (https://xenabrowser.net/datapages/), and the scRNA-seq data from HNSCC tumors were retrieved from GEO with accession code GSE103322. All processed data are available in the Figshare repository (https://figshare.com/articles/dataset/MulNet_Data/28106372). The normalized count matrix and metadata spreadsheet of our RNA-seq experiments are publicly accessible under accession number GSE248963 in the Gene Expression Omnibus (GEO). The raw sequencing reads are available in the NCBI Sequence Read Archive (SRA) under accession number PRJNA1012745. The source code and application of MulNet can be accessed from our GitHub repository, https://github.com/free1234hm/MulNet, under the GPL-3.0 license.
