# Supplementary material for: MulNet: a scalable framework for reconstructing intra- and intercellular signaling networks from bulk and single-cell RNA-seq data
Source: Brief Bioinform. 2025 Mar 17;26(2):bbaf081. doi: 10.1093/bib/bbaf081 (PMC11912874; doi:10.1093/bib/bbaf081)
Supplement: Supplementary_Materials_bbaf081 [file supplementary_materials_bbaf081.docx]

**Supplementary Methods**

**1. The probability of correlation between gene expression**

Correlation coefficients, such as Pearson, Spearman, Kendall, and cosine similarity, measure the strength of the linear relationship between two variables. However, they cannot determine the probability that two variables are correlated. We propose a novel probabilistic model to measure this probability for gene expression patterns.

Given an expression matrix with $m$ genes, the expression pattern of each gene is an $n$-dimensional vector. First, we calculate the pairwise Pearson correlation coefficients between genes. When $m$ is large enough, these correlation coefficients can be seen as random numbers between -1 and 1, whose quantity decreases as the value approaches 1 or -1. Based on these correlation coefficients, we can construct an approximate Gaussian distribution with a -1 to 1 range (Supplementary Figure S1). The mean of this Gaussian distribution is not necessarily zero, although it is often close to zero because in most cases, there is no significant difference between the numbers of positive and negative correlation coefficients.

The total area of this distribution is calculated as $S_{total}=CDF\left( 1 \right)-CDF(-1)$, where CDF stands for the cumulative distribution function. It is a function that gives the probability that a random variable will be less than or equal to a certain value. The area of a given correlation coefficient ($r$) is calculated as $S_{r}=CDF\left( \left| r \right| \right)-CDF(-\left| r \right|)$, and the significance probability of $r$ is calculated as the proportion of $S_{r}$ in the total area ($P_{r}=S_{r}/S_{total}$). To increase the distinction between low and high correlation coefficients, we define a minimum correlation coefficient threshold (0.4 by default). When the absolute correlation coefficient between two genes is lower than the minimum threshold, the probability of a correlation between them is defined as 0 ($\left| r \right|\in[-0.4,0.4]$). Otherwise, a minimum area ($S_{min}=CDF\left( 0.4 \right)-CDF(-0.4)$) is subtracted from both $S_{total}$ (the area shaded with stars in Supplementary Figure S1) and $S_{r}$ (the area shaded with slanted lines in Supplementary Figure S1). The final correlation probability is calculated as follows:

$$P(r)=\left\{ \begin{aligned} 0 \left| r \right|\leq0.4 \\ \frac{S_{r}-S_{min}}{S_{total}-S_{min}} \left| r \right|>0.4 \end{aligned} \right.$$

**2. Method evaluation**

**2.1 Evaluation pipeline using known modules**

Following the benchmark strategy proposed by Saelens [1], we calculated four metrics (recovery, relevance, recall, and precision) by comparing the identified modules with sets of known modules. If $G$ represents all genes, $M$ represents a set of known modules, $M^{'}$ represents a set of observed modules, $M(g)$ represents the modules that contain gene $g$, and $E(g,M)$ represents the set of genes that are included with $g$ in at least one module of $M$ (including $g$ itself), the precision is defined as follows:

$$Precision=\frac{1}{\left| G \right|}\sum_{g\in G} \left[ \frac{1}{\left| E(g,M^{'}) \right|}\sum_{g^{'}\in E(g,M^{'})} \frac{min(\left| M^{'}\left( g \right)\cap M^{'}\left( g^{'} \right) \right|,\left| M\left( g \right)\cap M\left( g^{'} \right) \right|)\times\Phi(g,g^{'})}{\left| M^{'}(g)\cap M^{'}(g^{'}) \right|} \right]$$

where $\Phi\left( g,g^{'} \right)=\frac{1}{\left| M^{'}\left( g,g^{'} \right) \right|}\sum_{m^{'}\in M^{'}\left( g,g^{'} \right)} \max_{m\in M\left( g,g^{'} \right)} Jaccard(m^{'},m)$. Recall is calculated in the same way but with $M^{'}$ and $M$ switched. Relevance is defined as follows:

$$Relevance=\frac{1}{\left| M^{'} \right|}\sum_{m^{'}\in M^{'}} \max_{m\in M} Jaccard(m^{'},m)$$

Recovery is calculated in the same way but with $M^{'}$ and $M$ switched. Third, we calculated the harmonic mean of precision, recall, relevance, and recovery to obtain an overall score. Finally, we normalized this score by dividing it by a benchmark score derived from a randomly permuted version of the known modules.

**2.2 Application to TCGA pan-cancer data**

We evaluated MulNet's performance using TCGA data from a pan-cancer study of 12 cancer types, comparing it with eight other bulk RNA-seq methods: ICA [2], agglomerative hierarchical clustering [3], FLAME [4], spectral clustering [5], densityClust [6], spectral biclustering [7], MERLIN [8], and GENIE3 [9]. The gene expression data and known modules were obtained from Zenodo (https://zenodo.org/records/1157938). Based on the original expression matrix, we performed principal component analysis (PCA) to extract the 30 most representative components for each gene. The processed expression matrix is available in the Figshare repository (https://figshare.com/articles/dataset/MulNet_Data/28106372). Detailed parameters for each method are provided below:

**2.2.1 MulNet**

We applied MulNet to integrate the processed gene expression data with three human reference interaction files—human TF-target.txt, human miRNA-target.txt, and human PPI.txt—available in the MulNet repository (https://github.com/free1234hm/MulNet). All other parameters were configured as follows:

Log normalize data: No

Max number of missing values: 0

Set missing values as: Min value

Min correlation coefficient: 0.4

Discount factor for the MDP: 0.995

Max number of modules: 10, 20, and 30

Min number of modules: 10, 20, and 30

**2.2.2 Existing methods**

Given that ICA performs better with large samples and other methods excel with smaller samples [1], we applied ICA to the original expression matrix and the other methods to the PCA-transformed matrix. This ensures that each method achieves its optimal performance. ICA was performed using the ‘ica_fdr’ function from Saelens et al. (https://github.com/saeyslab/moduledetection-evaluation), with parameters qvalcutoff = 0.1 and k = 10, 20, and 30. Spectral clustering was implemented using the SpectralClustering function from scikit-learn, with affinity = ‘nearest_neighbors’, n_neighbors = 20, and n_clusters = 10, 20, and 30. The FLAME algorithm was implemented based on the source code available at https://code.google.com/archive/p/flame-clustering/source/default/source. Agglomerative hierarchical clustering was performed using the R package ‘hclust’. The density-based clustering algorithm was implemented with the R package ‘densityClust’. The GENIE3 algorithm was applied using the GENIE3 R package. The MERLIN algorithm was implemented based on code from pages.discovery.wisc.edu/~sroy/merlin. To control for the impact of module number on evaluation metrics, we standardized the number of modules identified by each method to 10, 20, and 30. All other parameters were set to their default values.

**2.3 Application to scRNA-seq Data**

We applied MulNet and six existing methods (SCENIC [12], SCIMITAR [13], NLNET [14], SCODE [15], PIDC [16], and LEAP [17]) to 10 human cancer scRNA-seq datasets sourced from the Curated Cancer Cell Atlas (www.weizmann.ac.il/sites/3CA/). These datasets include those from breast (Chung et al., 2017), head and neck (Puram et al., 2017), prostate (He et al., 2021), colorectal (Li et al., 2017), liver (Ma et al., 2019), pancreatic (Moncada et al., 2020), lung (Song et al., 2019), hematologic, kidney (Young et al., 2018), and ovarian (Zhang et al., 2019) cancers.

For each dataset, we identified genes with missing values < 50% in malignant cells, resulting in varying numbers of genes across the 10 benchmark datasets (492, 692, 940, 1057, 1429, 1949, 2727, 3860, 4535, and 5190 genes). Based on the expression profiles of these gene sets in malignant cells, we constructed 10 malignant cell-specific expression matrices.

Since methods like SCENIC and PIDC are computationally intensive when analyzing expression matrices with large numbers of cells, we reduced the dimensionality of each expression matrix in two steps: 1) agglomerative clustering was used to group cells into 50 clusters, and 2) the average expression of each cell cluster was calculated. All processed expression matrices are available in the Figshare repository (https://figshare.com/articles/dataset/MulNet_Data/28106372).

**2.3.1 MulNet**

We constructed a multilayer network by integrating gene expression data with three human reference interaction datasets—human TF-target.txt, human miRNA-target.txt, and human PPI.txt—available in the MulNet repository (https://github.com/free1234hm/MulNet). The parameters were configured as follows:

Log normalize data: No

Max number of missing values: 0

Set missing values as: Min value

Min correlation coefficient: 0.4

Discount factor for the MDP: 0.995

Max number of modules: 10, 20, and 30

Min number of modules: 10, 20, and 30

**2.3.2 SCENIC**

SCENIC is implemented in both R and Python (https://github.com/aertslab/SCENIC) and employs the GENIE3 algorithm, a regression-tree-based method, to construct gene regulatory networks. While originally developed for bulk RNA-seq data, SCENIC has been applied to single-cell analysis. For this study, the number of candidate regulators randomly selected at each tree node (to determine the best split) was set to 1000 to generate a weighted adjacency matrix of the inferred network. Spectral clustering was then used to detect modules, with the ‘centers’ parameter set to 10, 20, and 30, respectively.

**2.3.3 NLNET**

NLNET is implemented in R and distributed as the nlnet package (https://cran.r-project.org/web/packages/nlnet/). It does not assume a specific functional form and can select from a large number of candidates. In NLNET, the correlation between two genes is defined as the distance based on a conditional ordered list. The minimum allowable value of the local false discovery cutoff for establishing gene links was set to 0.05. The parameter min.module.size was adjusted to 2, 3, and 5 to obtain 10, 20, and 30 clusters, respectively.

**2.3.4** **SCIMITAR**

SCIMITAR is a framework for inferring progressions from static single-cell transcriptomes by fitting continuous parameterizations of Gaussian mixtures to high-dimensional curves. It was implemented in Python (https://github.com/dimenwarper/scimitar). SCIMITAR first constructs a transition model from the metastable graph by fitting single-curve morphing Gaussian mixtures to specified states. This model is further refined using a ‘corpcor’ estimator with a shrinkage intensity of 0.05 until convergence. The n_cluster parameter in the plot_transition_clustermap function was set to 10, 20, and 30 to generate the corresponding clusters.

**2.3.5 PIDC**

PIDC is an algorithm designed to infer gene regulatory networks from single-cell data using multivariate information measures. It is implemented in Julia, with tutorials available at https://github.com/Tchanders/network_inference_tutorials. For the networks inferred by PIDC, the default threshold for filtering the highest-scoring edges was set to default (15%). Spectral clustering was then applied to detect modules, with the ‘centers’ parameter set to 10, 20, and 30, respectively.

**2.3.6 LEAP**

The LEAP algorithm, implemented in the R package LEAP, integrates scRNA-seq data with estimated pseudotime to construct gene co-expression networks that account for time delays. By sorting cells based on pseudotime, the algorithm calculates the maximum correlation across all possible time lags. This maximum correlation is then used as the edge weight in the gene co-expression network. Finally, a spectral clustering algorithm is applied to identify modules within the network, with the ‘centers’ parameter set to 10, 20, and 30, respectively.

**2.3.7 SCODE**

The SCODE algorithm is used to analyze scRNA-seq data from differentiating cells by integrating the transformation of linear ordinary differential equations (ODEs) with linear regression. The R source code for SCODE is available at https://github.com/hmatsu1226/SCODE. We set the length of the original expression dynamics vector $z$ to 4 and the number of optimization iterations to 100. In the adjacency matrix, directed edges with higher absolute values are considered to represent more reliable regulatory relationships. Finally, spectral clustering is applied to identify modules, with the ‘centers’ parameter set to 10, 20, and 30, respectively.

**3. Data collection and processing in case study 1**

To evaluate the effectiveness of MulNet in extracting biological insights from RNA-seq data, we applied it to the TCGA-COAD dataset from the UCSC Xena database. First, we divided patients into four clinical stages (I, II, III, and IV) according to the American Joint Committee on Cancer (AJCC) 8th edition TNM classification. This resulted in four stage-specific gene expression matrices containing data from 67, 161, 103, and 63 patients with stage I–IV COAD, respectively. Subsequently, we applied MulNet to integrate each expression matrix (after PCA) with three types of reference interactions: TF–target interactions from TRRUST [18], miRNA–target interactions from miRTarBase [19], and PPIs from HuRI [20]. The module detection parameters were set as follows:

- Log normalize data: No
- Max number of missing values: 0
- Set missing values as: Min value
- Min correlation coefficient: 0.4
- Discount factor for the MDP: 0.995
- Max number of modules: 50
- Min number of modules: 1

We constructed a ‘consensus network’ by integrating the signaling modules identified across stage I–IV COAD. In this network, nodes are connected only if they consistently participate in the same module across all stages. These connections can be based on various interaction types, including PPIs, TF–DNA interactions, miRNA–mRNA interactions, and, for genes lacking known interactions, coexpression relationships. Next, we performed network-based survival analysis on the consensus network to identify TF–target and miRNA–target pairs associated with overall survival in COAD patients. Our analysis utilized RNA-seq and survival data from TCGA-COAD patients. We employed a log-rank test with a significance threshold of 0.05 to determine statistical significance.

**4. Data collection and processing in case study 2**

Processed single-cell expression data (log2 TPM values) of malignant and nonmalignant cells from HNSCC tumors were downloaded from the GEO with the accession code GSE103322 [21]. We analyzed four gene expression matrices across four cell types, including 1112 cancer-associated fibroblasts (CAFs) and 1427 malignant cells from primary tumors and 310 CAFs and 788 malignant cells from lymph node metastatic tumors. For each expression matrix, we removed genes with more than 80% missing values and applied PCA to extract the 50 most representative components of each gene expression feature for MulNet analysis.

For each processed CAF expression matrix, we built a two-layered network focusing on TF–target interactions. From this network, we identified signaling modules and integrated them into a CAF-specific TF–target network. Additionally, for each processed tumor cell expression matrix, we constructed a three-layered network incorporating both ligand–TF and TF–target interactions. From this network, we identified signaling modules and integrated them into a ligand–TF–target network associated with tumor cells.

Subsequently, we applied network-based survival analysis to the TF–target network within CAFs and the ligand–TF–target network within tumor cells to identify TF–target pairs associated with overall survival in HNSCC patients. Since the GSE103322 dataset does not provide patient clinical outcomes, we employed RNA-seq and survival data from the TCGA-HNSCC cohort to identify survival-related TF–target pairs within the network generated based on GSE103322. This approach is based on the hypothesis that TF-target pairs significantly impacting HNSCC progression and patient survival exhibit robustness across datasets, despite batch effects and interindividual variability between the TCGA and GSE103322 cohorts. The second case study identified a substantial number of key regulators of HNSCC growth and metastasis, suggesting that the signaling network built from GSE103322 data is generalizable well to the TCGA-HNSCC cohort.

Finally, we assembled the survival-related TF–target pairs identified from the CAF and tumor cell networks, constructing a CAF–tumor cell crosstalk network (Figure 6a). This network reveals how CAF TFs regulate the expression of ligand molecules. These secreted ligands then bind to receptors on tumor cells and trigger intracellular transcriptional responses.

**5. Cell experiments**

**5.1 Cell lines and culture**

Human colon cancer cell line HCT116 was acquired from ATCC and cultivated in DMEM (12800-017, Invitrogen, Carlsbad, CA, USA) supplemented with 10% fetal bovine serum (FBS) (AB1001, ABBIOSCI, Seattle, WA, USA) and 1% penicillin-streptomycin (15140122, Invitrogen, Carlsbad, CA, USA) at 37 °C with 5% CO2. The media were changed every two days.

**5.2 Transfection**

HCT116 cells were plated in 12-well or 96-well plates on day 0. Twenty hours later, 50 nM miR-8485 mimics (GenePharma, Suzhou, China) were transfected using DMRIE-C transfection reagent (10459014, Thermo Fisher, Waltham, MA, USA) following the manufacturer’s protocol. Forty-eight hours after transfection or at the indicated times, the cells were collected for further experiments. The sequence of the miR-8485 mimic was 5'-CACACACACACACGUAU-3' (sense).

**5.3 Cell proliferation**

HCT116 cells were seeded into 96-well plates and cultured in DMEM supplemented with 10% FBS (8000 cells per well). miRNAs were transfected on day 1. Then, the cells were collected at 24 h, 48 h and 72 h after transfection and counted by CCK-8 reagent (CA1210, Solarbio, China) following the manufacturer’s protocol.

**5.4 Transwell Assay**

HCT116 cells were seeded in 6 cm plates and after transfected for 24 h to replate 0.5 million cells in the upper chamber of trans-well chamber (3422, Costar, Washington, DC, USA) in 200μL serum-free DMEM and 600 μL 15% FBS DMEM was added to the lower chamber and incubated for 48 h at 37°C. Nonmigratory cells were removed from the upper chamber by scraping with a cotton swab. The cells remaining on the lower surface of the insert were fixed with 4% paraformaldehyde (1004965000, Sigma, St. Louis, MO, USA) and stained with crystal violet (46364, Sigma, St. Louis, MO, USA). The number of migrated cells was counted by ImageJ.

**5.5 RNA extraction, RNA-seq, and bioinformatics analysis**

Total RNA was extracted following the standard TRIzol protocol (15596026, Invitrogen, Carlsbad, CA, USA). RNA libraries from three independent biological replicates were generated using the VAHTS Universal V8 RNA-seq Library Prep Kit for Illumina (NR605, Vazyme, Nanjing, China) according to the manufacturer’s instructions. Libraries were sequenced using an Illumina NovaSeq 6000 (Illumina, San Diego, USA). The original fluorescence image files obtained from the Illumina platform were transformed to short reads (raw data) by base calling, and these short reads were recorded in FASTQ format, which contains sequence information and corresponding sequencing quality information.

Using Fastp (version 0.23.1) [22], the raw sequenced reads were trimmed for adaptor sequences and masked for low-complexity or low-quality sequences. Paired-end clean reads were mapped to the reference genome hg38 using HISAT2 (v2.0.5). FeatureCounts (v1.5.0-p3) was used to calculate the read numbers mapped to each gene. Differential expression analysis of two conditions/groups (three biological replicates per condition) was performed using the DESeq2 R package (1.20.0). KEGG enrichment analysis of differentially expressed genes was implemented by the clusterProfiler R package. P-values were corrected for multiple testing using the Benjamini–Hochberg procedure.

**6. Animal experiments**

All animal experiments were performed according to protocols approved by the Institutional Animal Care and Use Committee (IACUC) at the Center for Experimental Animal Research of the Institute of Basic Medical Sciences (IBMS), Chinese Academy of Medical Sciences (CAMS). BALB/c nude mice were randomized into two groups. A total of 5 × 10^5^ HCT116 cells transfected with control or miR-8485 were injected into the dorsal flank of the mice. Tumor growth was measured weekly beginning on day 7 postimplantation. Tumor volumes were calculated using the following formula:

$$V=\frac{\pi}{6}\times length\times width\times thickness$$

The mice were sacrificed after 3 weeks, and the tumors were harvested.

**Supplementary Figures**


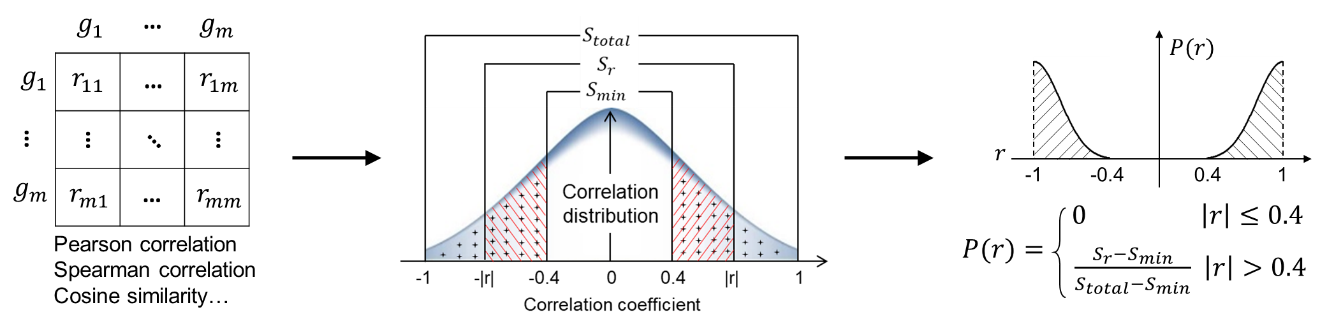


**Figure S1.** Procedure for calculating the probability of correlations between gene expression profiles using the cumulative distribution function of an approximate Gaussian distribution.

**
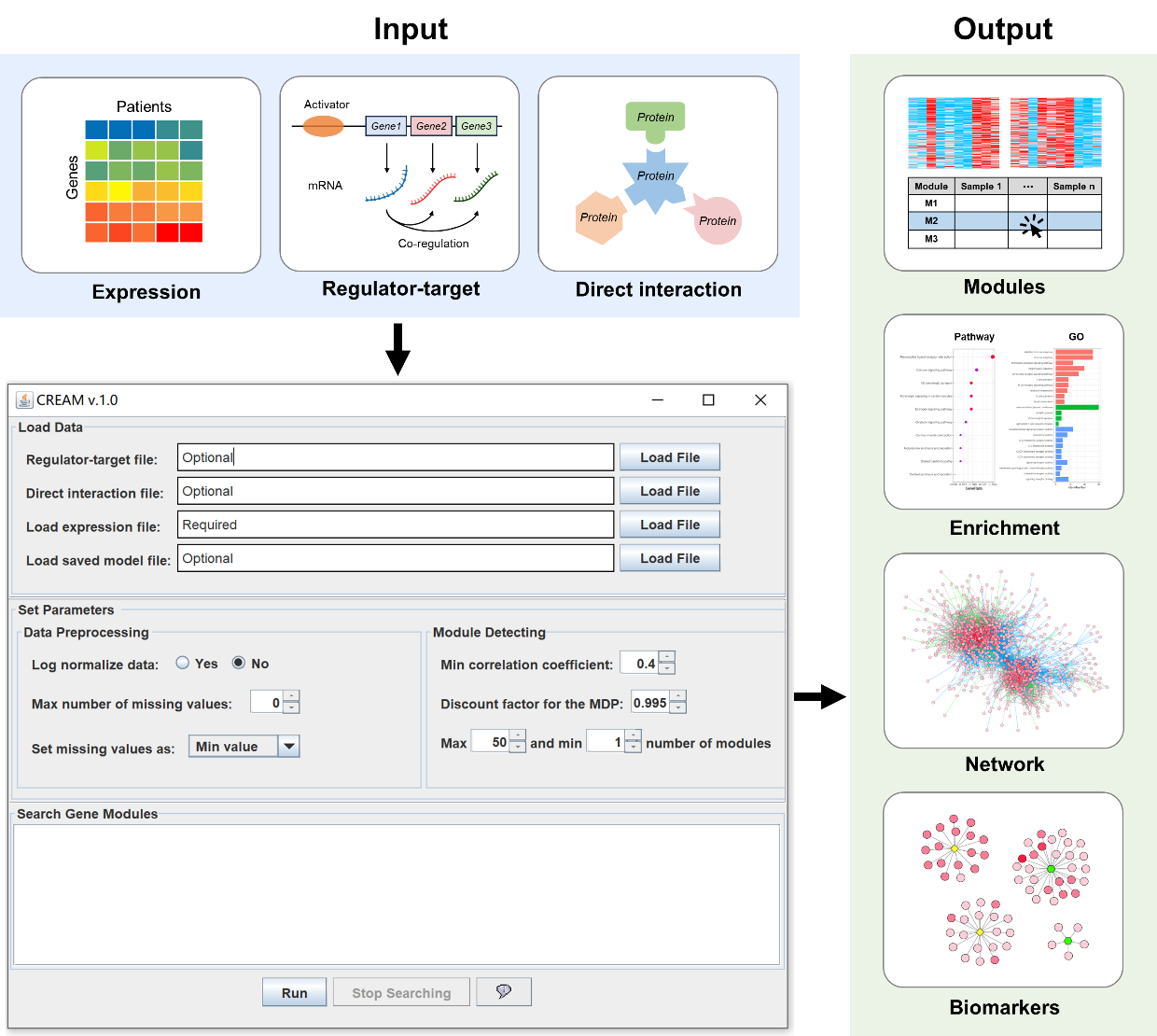
Figure S2.** Inputs (expression data and molecular interactions), main interface, and Interactive outputs of MulNet. MulNet visualizes identified modules with interactive tables and heatmaps, allowing users to select modules of interest for functional enrichment analysis, integrate all modules into a genome-wide hybrid network, and identify prognostic regulators.


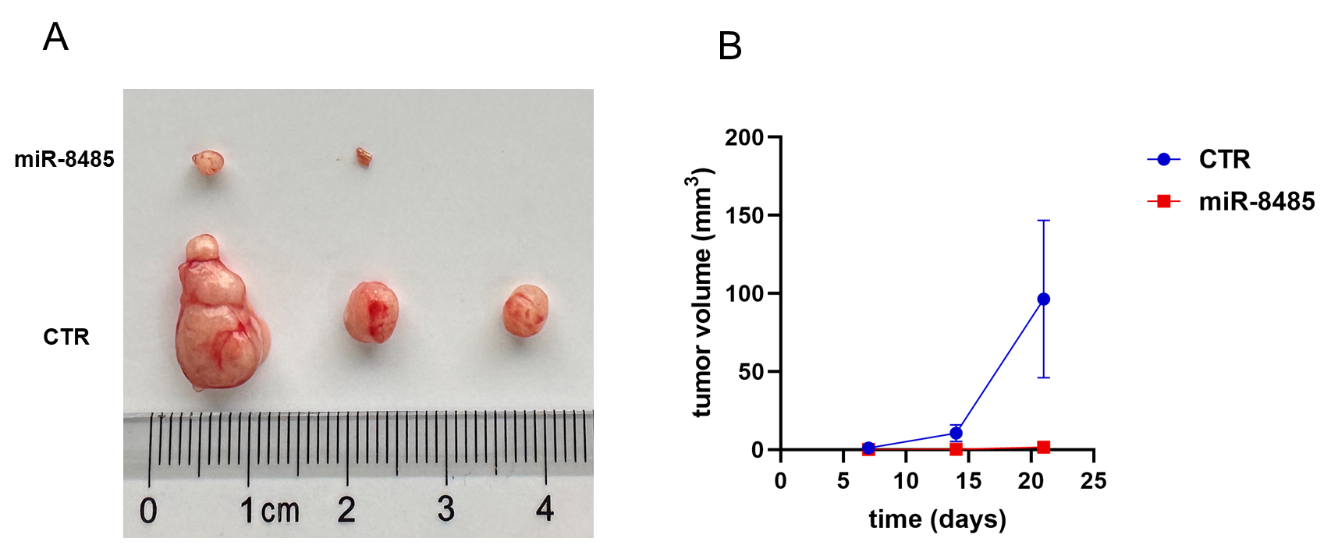


**Figure S3.** (A) Tumors harvested from mice that were injected with HCT116 cells transfected with either miR-8485 or a negative control (three weeks later). (B) Tumor volumes of the miR-8485 and control groups measured at day 7, 14, and 21 post-implantation.


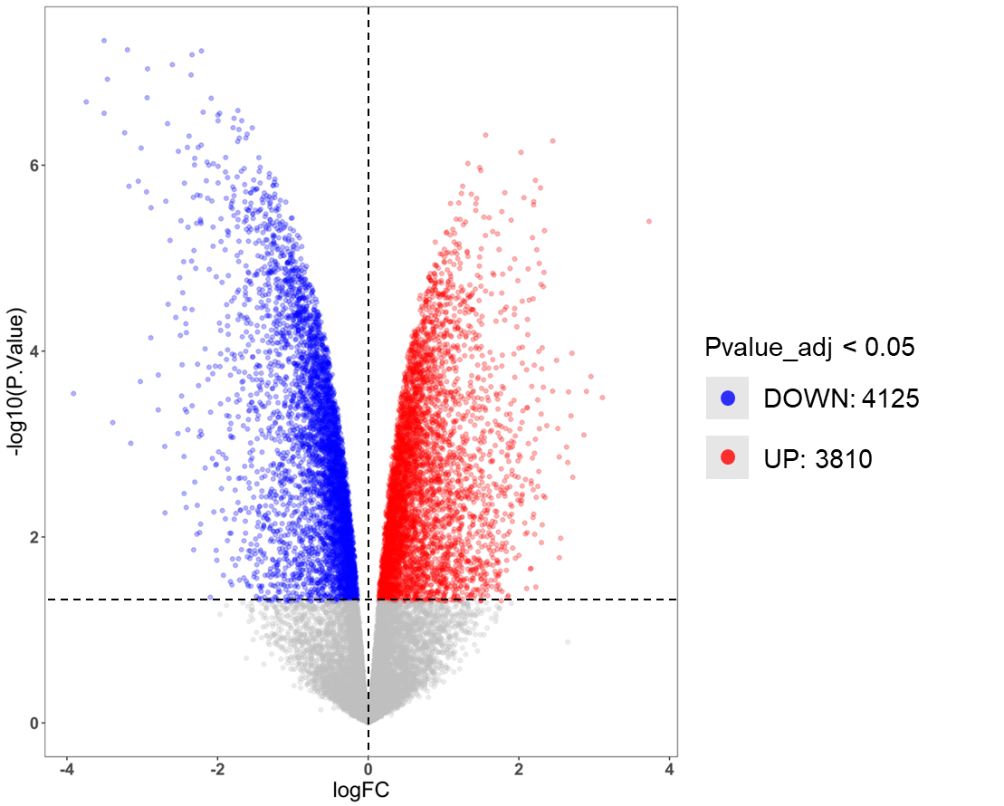


**Figure S4.** Volcano plot comparing miR-8485-transfected HCT116 COAD cells to the control group.


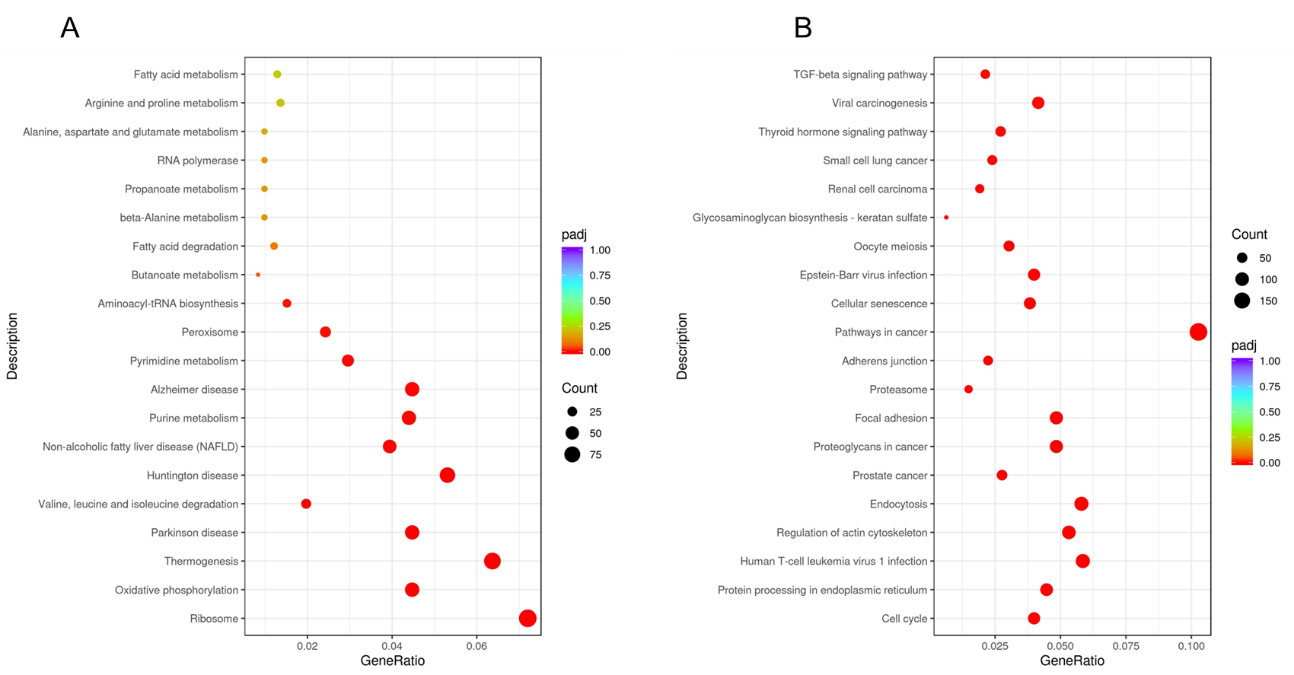


**Figure S5.** The top 20 pathways significantly enriched in the (A) up- and (B) down-regulated genes.

**REFERENCES**

1. Saelens, W., R. Cannoodt, and Y. Saeys, *A comprehensive evaluation of module detection methods for gene expression data.* Nat Commun, 2018. **9**(1): p. 1090.

2. Hyvarinen, A., *Fast and robust fixed-point algorithms for independent component analysis.* IEEE Trans Neural Netw, 1999. **10**(3): p. 626-34.

3. Langfelder, P. and S. Horvath, *WGCNA: an R package for weighted correlation network analysis.* BMC Bioinformatics, 2008. **9**: p. 559.

4. Fu, L. and E. Medico, *FLAME, a novel fuzzy clustering method for the analysis of DNA microarray data.* BMC Bioinformatics, 2007. **8**: p. 3.

5. Shi, J. and J. Malik, *Normalized cuts and image segmentation.* Pattern Analysis and Machine Intelligence, IEEE Transactions, 2000. **22**(8).

6. Rodriguez, A. and A. Laio, *Machine learning. Clustering by fast search and find of density peaks.* Science, 2014. **344**(6191): p. 1492-6.

7. Kluger, Y., et al., *Spectral biclustering of microarray data: coclustering genes and conditions.* Genome Res, 2003. **13**(4): p. 703-16.

8. Roy, S., et al., *Integrated module and gene-specific regulatory inference implicates upstream signaling networks.* PLoS Comput Biol, 2013. **9**(10): p. e1003252.

9. Huynh-Thu, V.A., et al., *Inferring regulatory networks from expression data using tree-based methods.* PLoS One, 2010. **5**(9).

10. Strimmer, K., *fdrtool: a versatile R package for estimating local and tail area-based false discovery rates.* Bioinformatics, 2008. **24**(12): p. 1461-2.

11. Langfelder, P., B. Zhang, and S. Horvath, *Defining clusters from a hierarchical cluster tree: the Dynamic Tree Cut package for R.* Bioinformatics, 2008. **24**(5): p. 719-20.

12. Aibar, S., et al., *SCENIC: single-cell regulatory network inference and clustering.* Nat Methods, 2017. **14**(11): p. 1083-1086.

13. Cordero, P. and J.M. Stuart, *Tracing Co-Regulatory Network Dynamics in Noisy, Single-Cell Transcriptome Trajectories.* Pac Symp Biocomput, 2017. **22**: p. 576-587.

14. Liu, H., et al., *Nonlinear Network Reconstruction from Gene Expression Data Using Marginal Dependencies Measured by DCOL.* PLoS One, 2016. **11**(7): p. e0158247.

15. Matsumoto, H., et al., *SCODE: an efficient regulatory network inference algorithm from single-cell RNA-Seq during differentiation.* Bioinformatics, 2017. **33**(15): p. 2314-2321.

16. Chan, T.E., M.P.H. Stumpf, and A.C. Babtie, *Gene Regulatory Network Inference from Single-Cell Data Using Multivariate Information Measures.* Cell Syst, 2017. **5**(3): p. 251-267 e3.

17. Specht, A.T. and J. Li, *LEAP: constructing gene co-expression networks for single-cell RNA-sequencing data using pseudotime ordering.* Bioinformatics, 2017. **33**(5): p. 764-766.

18. Han, H., et al., *TRRUST v2: an expanded reference database of human and mouse transcriptional regulatory interactions.* Nucleic Acids Res, 2018. **46**(D1): p. D380-D386.

19. Huang, H.Y., et al., *miRTarBase update 2022: an informative resource for experimentally validated miRNA-target interactions.* Nucleic Acids Res, 2022. **50**(D1): p. D222-D230.

20. Luck, K., et al., *A reference map of the human binary protein interactome.* Nature, 2020. **580**(7803): p. 402-408.

21. Puram, S.V., et al., *Single-Cell Transcriptomic Analysis of Primary and Metastatic Tumor Ecosystems in Head and Neck Cancer.* Cell, 2017. **171**(7): p. 1611-1624 e24.

22. Chen, S., et al., *fastp: an ultra-fast all-in-one FASTQ preprocessor.* Bioinformatics, 2018. **34**(17): p. i884-i890.
